# Pulmonary embolism due to an indefinite plug: 5-year follow-up

**DOI:** 10.1093/icvts/ivac202

**Published:** 2022-07-27

**Authors:** E Çil, K E Şahin

**Affiliations:** Department of Chest Diseases, Adiyaman University Education and Research Hospital, Adiyaman, Turkey; Department of Cardiology, Adiyaman University Education and Research Hospital, Adiyaman, Turkey

**Keywords:** Pulmonary embolism, Non-thrombotic, Indefinite plug

## Abstract

Thoracic computed tomography angiography revealed a filling defect with an internal air density in the lower lobar branch of the left pulmonary artery accompanied by pleural fluid, in a patient who applied with sudden onset chest pain and dyspnoea. The filling defect remained stable after anticoagulant treatment. No progression or complications were observed in 5-year follow-up. In pulmonary embolism that does not resolve despite adequate treatment, non-thrombotic sources, particularly foreign body, should be considered.

## INTRODUCTION

In most cases, pulmonary embolism may develop as a result of occlusion of pulmonary arteries with thrombus fragments originating from deep veins of lower extremities [1], or more infrequently due to non-thrombotic causes as in the present case.

## CASE DESCRIPTION

**Figure 1:**
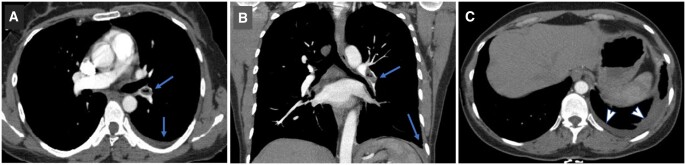
CT images at initial diagnosis, filling defect and pleural fluid are indicated by arrows, on axial plane (**A**) and on coronal plane (**B**). The atelectasis is indicated by arrowheads on axial plane (**C**).

A 49-year-old female was admitted to emergency service with a sudden onset of stabbing chest pain on the left side and shortness of breath. Heart rate was 110 bpm, blood pressure was 110/70 mmHg, oxygen saturation was 97% and body temperature was 36.5°C. Her past medical history was unremarkable except for lower extremity varicose vein embolization surgery with *n*-butyl cyanoacrylate (NBCA), 6 months ago. On laboratory, haemogram, C-reactive protein and biochemistry parameters were within normal limits and solely d-dimer was 3760 µg/l (0–500 µg/l). Filling defect including an air image in lower lobar branch of left pulmonary artery accompanied by minimal pleural fluid and atelectasis in left lower pulmonary lobe was detected on CT (Fig. 1A–C).

**Figure 2:**
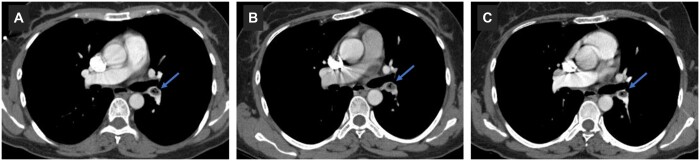
CT images at the 6th month control (**A**), the 1st year (**B**) and the 4th year (**C**) filling defect is indicated by arrow.

Transthoracic echocardography (TTE) and venous Doppler findings were normal except that left vena saphena magna was obliterated at the inguinal level. Invasive treatment was not considered since patient was haemodynamically and clinically stable after oxygen and heparinization. She was discharged with warfarin on the 6th day of her hospitalization, but we switched to apixaban 5 mg bid, due to nosebleed and bruises with an INR of 2.6. Thorax computed tomography angiography (CTA) on the 6th-month control revealed no significant change in the size of the plug, except the pleural fluid was completely cleared away (Fig. 2A). On 1st- and 4th-year CTA scans, the size of plug did not change significantly and the image of air inside the plug persisted (Fig. 2B and C). Apixaban was discontinued on the 4th-year control, and considering the benefits in preventing recurrent thromboembolic events, we decided to continue with aspirin [1]. There was no change on chest X-ray and TTE at the fifth-year control following 1-year ASA as well.

## DISCUSSION

The most identified non-thrombotic sources of pulmonary embolism are fat, foreign body, air, tumour cells, infected vegetations, parasites and amniotic fluid [2]. The most common causes of embolism due to foreign bodies reported in the literature are intravenous devices and ruptured catheters and materials. Rarely, it may occur due to penetrating injuries caused by industrial accidents, explosions or shrapnel fragments [3]. Our case did not have a history of bone fracture due to tumour, pregnancy or trauma, and she had no complaints in favour of parasitic infection. Vascular tumour was not considered due to the stable course of thrombus in the 5-year follow-up. There is no definitive consensus regarding the removal of foreign bodies that cause pulmonary embolism; however, it is widely accepted that endovascular intervention should be considered first and surgery should be performed in case of failure [4]. In a study of 140 patients who underwent endoscopic sclerotherapy injection with NBCA, it was reported that the affected patients were either asymptomatic or mildly symptomatic, and no deaths directly related to this complication were reported [5]. Also, in our case, no significant complication was observed in the 5-year follow-up.

In thorax CTA, thrombotic-induced pulmonary embolisms appear round, whereas NBCA-induced emboli have a more tubular or nodular appearance and also embolisms due to NBCA are denser compared to the surrounding tissues except for bone tissue. In our case, NBCA, which was used in varicose embolization 6 months ago, might had organized with fibrin in course of time and the air given mistakenly during injection might had facilitated the rupture from the main body of glue and resulted in embolism. This might explain why the embolic plug in our case had a density similar to the surrounding tissue, except for some air image in its centre. However, since endovascular intervention and/or surgery were not considered, a definitive pathological diagnosis could not be made in the present case.

Removal of the embolic material is usually the treatment of choice in foreign body-related embolisms. However, there is no consensus in such stable cases when there is no need for removal. In our case, 6-month time gap between the embolic event and the injection procedure increased d-dimer levels and the near isodensity of the plug on CTA, all favoured initiation of an anticoagulation. How long to continue with anticoagulation or whether aspirin has benefits in non-thrombotic embolism are still the questions awaiting definite answers. In addition, if no intervention is required initially, late removal attempts should be omitted due to the risk of local adhesions and rupture. We believe that once a satisfactory diagnosis is made, annual follow-up based on symptoms and physical findings will be sufficient in stable cases.

In conclusion, patient history plays an important role in the diagnosis of pulmonary embolism due to foreign bodies. Non-thrombotic embolic sources should be kept in mind in pulmonary embolism cases who keep stable, either clinically or radiographically, despite appropriate treatment.

**Conflict of interest:** none declared.

## Reviewer information

Interactive CardioVascular and Thoracic Surgery thanks Hung-Lung Hsu, Jürg Schmidli and the other, anonymous reviewer(s) for their contribution to the peer review process of this article.
